# Hybrid FDG-PET/MR imaging of chronic osteomyelitis: a prospective case series

**DOI:** 10.1186/s41824-019-0055-5

**Published:** 2019-05-07

**Authors:** Dennis Jan Willem Hulsen, Jan Geurts, Jacobus J. Arts, Daan Loeffen, Cristina Mitea, Stefan Adrian Vöö

**Affiliations:** 1 Department of Orthopaedic Surgery, Research School CAPHRI, Maastricht University Medical Centre, Maastricht, The Netherlands; 2 MICT Department, Jeroen Bosch Ziekenhuis, ‘s-Hertogenbosch, The Netherlands; 3 Department of Radiology and Nuclear Medicine, Maastricht University Medical Centre, Maastricht, The Netherlands; 4 Institute of Nuclear Medicine, University College Hospital, London, UK

**Keywords:** Osteomyelitis, PET/MRI, Diagnosis, Operative planning

## Abstract

**Background:**

Magnetic resonance imaging (MRI) and 2-[18F]-fluoro-2-deoxy-D-glucose (FDG) positron emission tomography paired with computed tomography (PET/CT) are two commonly used imaging modalities in the complicated diagnostic workup of osteomyelitis. Diagnosis using these modalities relies on, respectively, anatomical (MRI) and metabolic (PET) signs. With hybrid PET/MRI being recently available, our goal is to qualitatively compare hybrid FDG PET/MRI to FDG PET/CT in the diagnosis and operative planning of chronic osteomyelitis.

**Methods:**

Five patients with suspected chronic osteomyelitis in an extremity underwent an ^18^F-FDG single-injection/dual-imaging protocol with hybrid PET/CT and hybrid PET/MR. Images and clinical features were evaluated using a standardized assessment method. Standardized uptake value (SUV) measurements were performed on all images. Concordant and discordant findings between PET/MRI and PET/CT were analysed.

**Results:**

The consensus diagnoses based on PET/MRI and PET/CT images were identical for all five patients. One discrepancy between PET/MRI and PET/CT was found in the assessment of the features in one patient. PET signal intensities and target-to-background ratios were on average highest for PET/MRI. On PET/MRI, the location of infection based on FDG uptake could clearly be correlated with certain soft tissue structures (oedema, fluid collection, or muscle), which is paramount for surgical planning.

**Conclusions:**

In the presented cases, FDG PET/MRI led to the same diagnosis and provided at least the same diagnostic information as PET/CT. PET/MRI was able to provide additional soft-tissue information for the physician planning treatment. Because of this, we suggest that PET/MRI could be used for osteomyelitis diagnosis and treatment planning.

## Introduction

Healthy bone is relatively resistant to infection, but haematogenous spread of bacteria, a contiguous source of infection, or trauma may lead to the onset of osteomyelitis. Osteomyelitis is defined as an infection of the bone and/or bone marrow. The most widely used classification system for osteomyelitis is the Cierny-Mader classification, which describes the bone involvement as well as the host status (Cierny et al. 2003). The infection can be caused by various microorganisms, of which gram-negative anaerobic bacteria are the most common cause (Sheehy et al. 2010). The lower extremities are the most regularly affected body parts (Kaim et al. 2002). Osteomyelitis is characterized by progressive inflammatory destruction and new apposition of the bone, leading to bone deformity and function loss. Its clinical manifestations are heterogeneous depending on patient characteristics, specific causative microorganism, anatomic area, the route of contamination, host factors, and comorbidities. One of the greatest challenges of osteomyelitis is to make an opportune diagnosis to provide adequate treatment, because prompt appropriate therapy may prevent both bone necrosis and bone function loss. Acute osteomyelitis can often be treated with systemic antibiotic administration but could progress into chronic osteomyelitis if treatment fails. Chronic osteomyelitis treatment frequently requires surgical debridement in combination with local and systemic administration of antibiotics (Parsons and Strauss 2004). Chronic osteomyelitis is primarily a clinical diagnosis, although the clinical picture may be confusing. Clinical follow-up combined with histopathology and microbiology is the gold standard diagnostic modalities, but this requires surgical intervention (Lew and Waldvogel 2004). For a preliminary clinical diagnosis, serum markers of inflammation, clinical evaluation, and imaging are routinely used (Vaidyanathan et al. 2015; Kumar et al. 2008). However, serum markers lack sensitivity in low-grade infections and are more suitable for trend analysis in those cases (Harris et al. 2011; Husain and Kim 2002).

Several imaging modalities have been used in the evaluation of suspected (chronic) osteomyelitis, but none can definitively confirm the presence or absence of infection (Pineda et al. 2006). Conventional X-ray imaging is usually the first modality to be used in the diagnostic process, but it is nonspecific and only able to depict latent effects of osteomyelitis on bone anatomy. Cross-sectional imaging with computed tomography (CT) shows excellent spatial resolution but is limited in osteomyelitis imaging by its poor soft tissue contrast. Soft tissues that are involved in osteomyelitis such as muscular structures, bone marrow, and oedema can be imaged distinctively using magnetic resonance imaging (MRI). For that reason, MRI is today considered of additional value in the diagnosis of osteomyelitis (Lee et al. 2016). It is preferable over CT because of its excellent anatomical detail, high sensitivity, and better ability to image bone marrow oedema and soft tissue involvement. This modality has a reported sensitivity of 70–90% and gives an excellent anatomic delineation of the infected or oedematous area and the surrounding soft tissue (Termaat et al. 2005; van der Bruggen et al. 2010). Specificity of MRI is relatively low for osteomyelitis (40–80%), and the images can lead to an overestimated severity and extent of infection (Lee et al. 2016; Termaat et al. 2005).

Whilst the aforementioned modalities are used to depict anatomical signs of osteomyelitis, nuclear medicine techniques can image specific physiological mechanisms (Love and Palestro 2016). Commonly applied nuclear medicine techniques in osteomyelitis workup include three-phase bone scintigraphy (possibly complemented by singe-photon emission tomography (SPECT)), in vitro-labelled leukocyte scans, and 2-[18F]-fluoro-2-deoxy-D-glucose (FDG) positron emission tomography (PET) scans. Bone scintigraphy is a traditional radionuclide technique that is founded on hydroxyapatite deposition. It is sensitive (83% sensitivity) in detecting osteomyelitis but not specific (45%) (Wang et al. 2011). Labelled leukocyte imaging is based on the recruitment of leukocytes by infections. This makes the method relatively specific (88%), with a sensitivity of 74% (Wang et al. 2011). The method however requires an intensive labelling process and is not available in every nuclear medicine facility. FDG PET images are based on metabolic activity. FDG is a glucose analogue that is transported to and accumulated in high glucose-demanding cells, such as active leukocytes at an infection. FDG PET is widely available and has the most preferable sensitivity (94.6%) and specificity (91.5%) compared to the aforementioned techniques (Termaat et al. 2005; Wang et al. 2011).

Hybrid imaging development combining PET and SPECT with CT has significantly advanced nuclear medicine. CT has added anatomical reference, novel attenuation correction methods, and to some extent diagnostic information to PET and SPECT. In a series of 215 patients, Wenter et al. showed that PET/CT had a higher sensitivity and specificity as compared to stand-alone PET in osteomyelitis diagnosis (Wenter et al. 2016). Recently, hybrid PET/MRI has become commercially available, combining metabolic imaging on PET with the high-resolution anatomical imaging and soft tissue contrast from MR. Because of the superiority of MRI compared to CT in osteomyelitis diagnosis, we expect that hybrid PET/MRI could be even more sensitive and specific in osteomyelitis diagnosis than PET/CT. Moreover, imaging is essential to the clinician planning surgical treatment. We expect MRI to provide additional soft tissue anatomical reference and soft tissue and oedema diagnostic information, thereby improving surgical planning.

The goal of this study is to qualitatively compare FDG PET/MRI to FDG PET/CT in both the diagnosis and operative planning of chronic peripheral osteomyelitis. A prospective case series is presented to illustrate the potential benefit of hybrid PET/MRI in osteomyelitis imaging.

## Methods

### Patient population

In this prospective single-centre study, patients referred to FDG PET/CT imaging for evaluation of suspected chronic osteomyelitis of an extremity were recruited between November 2016 and June 2017 at the Maastricht University Medical Centre. The specific diagnosis for these patients with conventional imaging and/or clinical examination had remained inconclusive, but there was a high suspicion for chronic osteomyelitis. For all patients, surgical intervention was indicated unless PET/CT would provide a negative result. Patients were excluded if they were under 18 years old, demented, pregnant, nursing, had previous surgery or implants in the region of interest, or had contra-indications for MRI. Of the six eligible patients in the aforementioned timeframe, five patients gave informed consent. The resulting five subjects consisted of three men and two women, ages 26, 48, 49, 53, and 64 at the time of acquisition. This study was approved by the institute’s board of directors with positive advice of the local medical-ethical review committee (reference METC 16-4-150.1/ab, Maastricht University Medical Centre, Maastricht, Netherlands). Our institute is a designated referral centre for osteomyelitis treatment.

### Image acquisition

The patients underwent a single-injection/dual-imaging protocol with ^18^F-FDG using a standard procedure. A dose of 2 MBq/kg FDG was injected intravenously. The radiopharmacon was acquired from a commercial radiopharmacy (GE Healthcare Radiopharmacy, Eindhoven, Netherlands). Patient blood glucose levels were confirmed to be < 10 mmol/l prior to scanning. Scanning commenced 56+/− 5 min after injection on the first modality and 123+/− 11 min after injection on the second modality. In four cases, the PET/CT was acquired first and in one case PET/MRI was acquired first.

Hybrid PET/CT scans were acquired with a Philips Healthcare Gemini TF system. The scanner has an axial field of view (FOV) of 18 cm, 90-cm ring diameter, The Association of Electrical Equipment and Medical Imaging Manufacturers (NEMA)-specified spatial resolution near FOV centre of 4.3 mm, and sensitivity near FOV centre of 7000 cps/MBq. The patients were scanned in supine position with 2 min per bed position. Acquired images were corrected for scatter, CT-based attenuation, and point spread function and reconstructed in a 344 × 344 matrix with OSEM time-of-flight-based iterative reconstruction, three iterations and 21 subsets with a 4-mm Gauss filter. A standard CT scan was acquired with 120-kV tube voltage.

Hybrid PET/MRI scans were acquired with a Siemens Healthcare Biograph mMR system. The scanner has an axial field of view (FOV) of 25.8 cm, 65.6-cm ring diameter, a NEMA-specified spatial resolution near FOV centre of 4.4 mm, and sensitivity near FOV centre 13,200 cps/MBq. PET scans were acquired for a single bed position, with duration extended to the duration of MRI acquisition, ranging from 10 to 20 min. Attenuation maps were obtained by a four-tissue (air, soft tissue, fat, and lung) Dixon-volume-interpolated mode (Martinez-Möller et al. 2009). All attenuation maps were qualitatively examined visually during the scanning process (Ladefoged et al. 2014). Acquired images were corrected for scatter, attenuation, point spread function, and time-of-flight and reconstructed in a 344 × 344 matrix with OSEM iterative reconstruction, three iterations and 21 subsets with a 4-mm Gauss filter. Standard MRI sequences were acquired: T1 turbo spin echo (TSE), T2 TSE Dixon, proton density TSE, and T2 with fat suppression by short TI inversion recovery (STIR) (Kapoor et al. 2007). Acquisitions were typically made in two anatomical planes, based on the anatomical location of the suspected lesion. A flexible MRI receiver coil was used that is specifically designed for use in PET/MRI.

Although it would be standard for regular osteomyelitis-indicated MRI, no intravenous gadolinium contrast-enhanced MRI was performed in this study for ethical considerations, as it would be an additional burden for the subjects compared to a routine PET/CT scan.

### Image assessment

All PET scans were assessed by an experienced nuclear medicine physician (SV), and MRI and CT scans were assessed by an experienced radiologist (DL). Both specialists were specifically trained for musculoskeletal imaging. Assessments were performed twice by the same specialist, at least 2 months apart to improve intra-observer reliability. For every patient, a list of features that the surgeon needs to establish for proper pre-operative planning was assessed (Govaert et al. 2017):

- Bone marrow involvement: for classification according to the Cierny-Mader system and to plan the extent of surgical debridement
- Soft tissue extent: for classification according to the Cierny-Mader system and to plan the extent of surgical debridement
- Cortical disruptions: for classification according to the Cierny-Mader system, to visualize mechanical integrity, and for treatment planning
- Presence of sequestra: for surgical planning, as an unresected sequestrum is a breeding ground and high-risk factor for a recurrent infection
- Presence and visibility of fistulae: to correspond the location of the external fistula end to the location of the osteomyelitic lesion for surgical planning

Additionally, the uptake of ^18^F-FDG was quantified in two ways, to objectively evaluate discrepancies between PET/MRI and PET/CT images:

1. The local maximum standardized uptake value (SUV) intensity of the lesional FDG accumulation (SUV_max_)
2. A target to background ratio (TBR), i.e. the lesional SUV_max_ divided by the average SUV in an internal reference region close to the lesion with a visually normal intensity on PET images and normal appearance on CT or MR images, respectively

After initial separate assessment, the imaging specialists together performed a consensus reading of PET/CT and PET/MR images and a combined conclusion was drawn. The value of both hybrid images for diagnosis and operative planning were discussed amongst the imaging specialists and the referring orthopaedic surgeon (JG). The Cierny-Mader classification was performed based on PET/CT and PET/MRI by the orthopaedic surgeon. Definitive diagnosis was made after surgery based on bone microbiology (five tissue samples cultivated for 2 weeks) and the complete perioperative and clinical picture.

## Results

### Patient 1

A 53-year-old male heavy smoker with a normal posture had a history of osteomyelitis in his left tibia since 1980. The patient recently had intermittent flare-ups with local symptoms around the site of former proximal fixator screws. He had been on analgesics for 4 years and had pus-producing fistula at his right proximal tibia when he consulted the orthopaedic surgeon. His lab values showed an erythrocyte sedimentation rate (ESR) of 26 mm/h (age- and gender-specific normal level < 20 mm/h), leukocyte count (WBC) of 8.8 × 10^9^/l (normal range 4–10 × 10^9^/l), and C-reactive protein concentration (CRP) of 13 mg/l (normal level < 10 mg/l). Conventional radiology was inconclusive about chronic osteomyelitis.

**Fig. 1:**
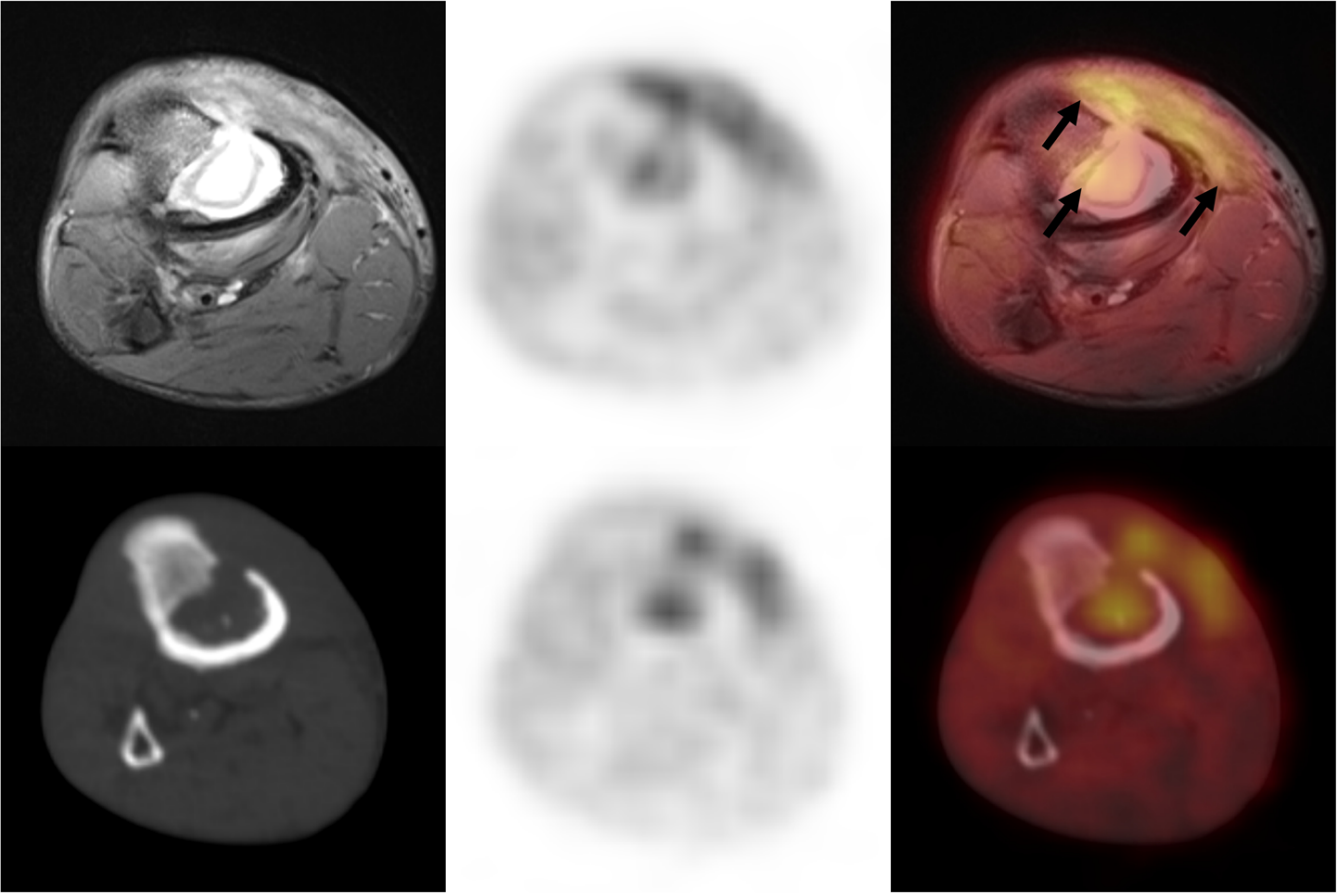
Reconstructed axial images of the affected region (left tibia) from patient 1. Top row, left to right: T2-weighted MRI, PET component of PET/MRI, and hybrid PET/MRI overlay. Bottom, from left to right: CT, PET component of PET/CT, and hybrid PET/CT overlay. Black arrows denote on hybrid PET/MRI, and the area of high FDG uptake can clearly be demarcated by soft tissue features that are depicted by MRI

Axial slices of all imaging modalities of the affected region of this patient are displayed as an example in Fig. 1. The presence of a fistula was indeterminate on CT and on the PET component of PET/MR but could be confirmed on MRI and the PET component of PET/CT (Table 1). The assessors noticed a discrepancy in the extent of the high signal on PET and high intensity on T2-weighted MRI. The MR images showed a more proximally extended high signal on T2-weighted images, which is indicative of oedema that did not show increased PET tracer uptake. The PET/MRI SUV_max_ TBR in this region of MRI-positive bone oedema was 1.7, from which it was concluded that this local bone marrow was not actively infected. Lesion PET TBR was similar for PET/CT and PET/MR. PET/CT showed higher SUV_max_: 4.7 compared to 3.2 for PET/MR. PET/CT and PET/MRI resulted in the same diagnosis of osteomyelitis Cierny-Mader type 3, class B. The patient refused immediate surgical intervention, making a definitive diagnosis unavailable. The clinical follow-up and imaging led to the clinical diagnosis of osteomyelitis.

**Table 1:** Consensus results for patient 1

	Bone marrow	Soft tissue	Cortex disruption	Sequester	Fistula	SUV_max_	PET TBR
PET/CT	A	A	Y	N	Y/I (Y)	4.7	5.2
PET/MR	A	A	Y	N	I/Y (Y)	3.2	5.3

*A* affected, *Y* yes, *N* no or not affected, *I* indeterminate

### Patient 2

A 49-year-old non-smoking obese female had a history of an osteotomy of the right first metatarsal bone in 2001. A painful pus-producing fistula appeared at this site in 2013, for which she received a steroid treatment with the diagnosis of non-septic arthritis. After 3 years of intermittent fistula opening, pain, and an arthrodesis of the MTP-1 joint, she was referred to our department. She had no signs of redness, warmth, or swelling but was still in pain. Lab values showed ESR 40 mm/h (age- and gender-specific normal level < 20 mm/h), WBC 5.9 × 10^9^/l, and CRP 13 mg/l, and conventional radiology was inconclusive about osteomyelitis.

The presence of an active intra- and extraosseous infectious process was confirmed on both hybrid modalities (Table 2). Soft tissue involvement was more pronounced on PET/MRI than on PET/CT, and PET/MRI revealed a fistula that could not be recognized on PET/CT images. Similar to patient 1, MRI (both T1 and T2) showed a larger extent of the oedema compared to PET. The PET/MRI SUV_max_ in the MRI positive region was 1.0, from which it was concluded that the region was not actively infected. PET signal TBR was considerably higher for PET/MRI (9.7) compared to PET/CT (4.8). Absolute SUV_max_ was 5.3 in PET/MRI and 4.8 in PET/CT. PET/CT and PET/MRI resulted in the same diagnosis of osteomyelitis Cierny-Mader type 3, class B. Microbiologic cultures for this patient were negative, but the perioperative image was very suggestive for osteomyelitis. Furthermore, the infection site was very small, increasing the likelihood of a false negative. Based on multidisciplinary counsel, a definitive diagnosis of osteomyelitis was made.

**Table 2:** Consensus results for patient 2

	Bone marrow	Soft tissue	Cortex disruption	Sequester	Fistula	SUV_max_	PET TBR
PET/CT	A	I/A (A)	Y	N	N	5.3	4.8
PET/MR	A	A	Y	N	I/Y (Y)	6.8	9.7

*A* affected, *Y* yes, *N* no or not affected, *I* indeterminate

### Patient 3

A 63-year-old non-smoking obese male had a traffic accident-related closed fracture in his left femur in 2002. He experienced recurrent inflammation signs at the site of a former intramedullary nail. Surgical debridement and gentamicin-loaded PMMA-bead implantation could not prevent recurring infection signs including a pus-producing fistula. Lab values showed ESR 32 mm/h (age- and gender-specific normal level < 20 mm/h), WBC 9.5 × 10^9^/l, and CRP 9 mg/l, and conventional radiography was suggestive for osteomyelitis.

The consensus reading of both PET/MRI and PET/CT images resulted in a diagnosis of osteomyelitis with affected soft tissue (Table 3). Both hybrid modalities showed intense FDG accumulation caudally in and around the femur. On the MR images, intramedullary involvement was assessed more extended than on PET.

**Table 3:** Consensus results for patient 3

	Bone marrow	Soft tissue	Cortex disruption	Sequester	Fistula	SUV_max_	PET TBR
PET/CT	A	A	Y	N	Y	10.0	16.7
PET/MR	A	A	Y	N	Y	11.2	22.4

*A* affected, *Y* yes, *N* no or not affected, *I* indeterminate

MR images depicted an area of high signal adjacent to the infectious processes that were equivocal for fluid collection or oedema. These regions could clearly be assessed as infected on PET with a SUV_max_ ranging from 3.5 to 8.6. PET signal TBR was higher for PET/MRI (22.4) compared to PET/CT (16.7), and absolute SUV_max_ was similar: 11.2 in PET/MRI and 10.0 in PET/CT images. PET/CT and PET/MRI resulted in the same diagnosis of osteomyelitis Cierny-Mader type 3, class B. Microbiology results were positive for *Staphylococcus aureus*, leading to a definitive diagnosis of osteomyelitis.

### Patient 4

A 26-year-old non-smoking male with a normal posture had received surgical debridement treatment for osteomyelitis in 2012. The patient who also suffered from sickle-cell anaemia experienced intermittent local fistulation and inflammation-related malaise. No conventional imaging was acquired. Lab values showed ESR 2 mm/h (age- and gender-specific normal level < 15 mm/h), WBC 9.5 × 10^9^/l, and CRP 75 mg/l.

Patient 4 showed the highest PET TBR of this series: 46.7 and 26.3 for PET/MRI and PET/CT, respectively (Table 4). The SUV_max_ of the infection was 10.5 on PET/CT and 14.0 on PET/MR.

**Table 4:** Consensus results for patient 4

	Bone marrow	Soft tissue	Cortex disruption	Sequester	Fistula	SUV_max_	PET TBR
PET/CT	N/A (N)	A	Y	Y	Y/I (Y)	10.5	26.3
PET/MR	N/A (N)	A	Y	Y	Y	14.0	46.7

*A* affected, *Y* yes, *N* no or not affected, *I* indeterminate

**Fig. 2:**
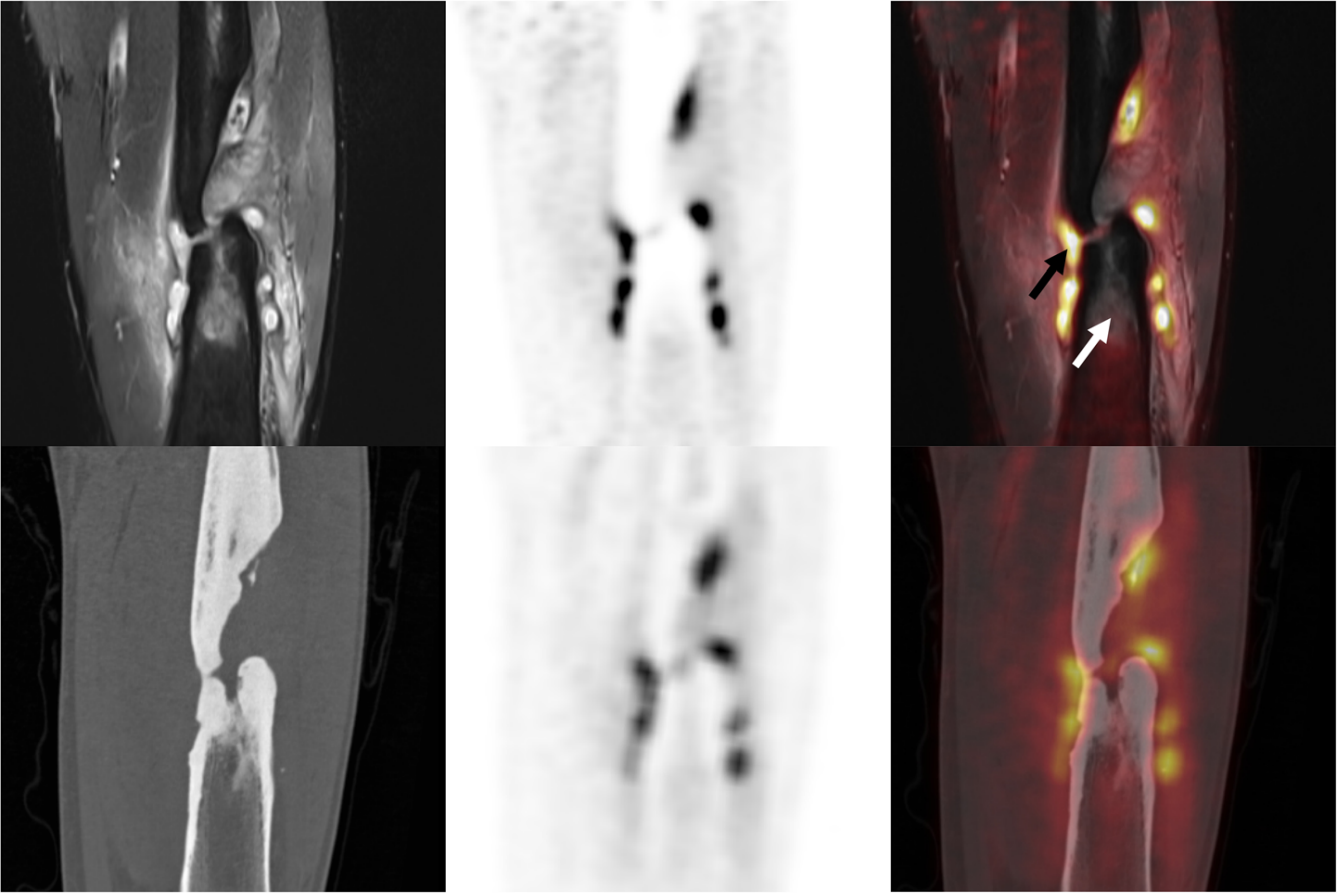
Reconstructed coronal images of the affected region (left femur) from patient 4. Top row, left to right: T2-weighted MRI, PET component of PET/MRI, and hybrid PET/MRI overlay. Bottom, from left to right: CT, PET component of PET/CT, and hybrid PET/CT overlay. Black arrow denotes PET/MRI is able to correlate the location of high FDG uptake with high intensity on T2-weighted MRI. White arrow denotes T2-weighted MRI shows intramedullary oedema that does not show increased FDG uptake. The features at both arrows are very influential information for a physician planning treatment

The bone marrow was assessed active based on CT and MR images, but this was contradicted by the PET images. This lack of intramedullary FDG uptake was depicted most profoundly by PET/MR imaging (Fig. 2). PET/MRI SUV_max_ for the bone marrow was 1.2 in the affected side, and 0.7 in the contralateral side. In the consensus reading, the bone marrow was assessed to be physiologically activated by the infection but not actively infected itself. PET imaging depicted a ring of soft tissue infection around the bone. In the hybrid PET/MR images, the infectious process was found to be clearly delineated by the muscle fascia. PET/CT and PET/MRI resulted in the same diagnosis of osteomyelitis Cierny-Mader type 3, class B. This diagnosis was confirmed by microbiological cultures that revealed the presence of Methicillin-resistant *Staphylococcus aureus* and *Salmonella Enteritidis*.

### Patient 5

A 48-year-old heavy-smoker obese female had chronic problems at a distal tibia congenital amputation stump that had required surgical intervention at childhood. She recently experienced a sudden soft tissue breakdown. Lab values showed ESR 16 mm/h (age- and gender-specific normal level < 20 mm/h), WBC 8.2 × 10^9^/l, and CRP 2 mg/l. Conventional radiology was inconclusive about osteomyelitis.

Diffuse FDG accumulation was found in soft tissue, but no accumulation was present in the bone (Table 5). Both modalities ruled out osteomyelitis. The extent of the soft tissue defect was interpreted clearest on PET/MR. This was the only patient that was scanned with PET/MRI prior to PET/CT. PET/CT showed the highest SUV_max_ (3.7 versus 2.3 for PET/MR) and TBR (7.4 versus 5.8). The perioperative image was suggestive for osteitis and could definitively rule out osteomyelitis. Microbiological results were positive for coagulase-negative Staphylococcus.

**Table 5:** Consensus results for patient 5

	Bone marrow	Soft tissue	Cortex disruption	Sequester	Fistula	SUV_max_	PET TBR
PET/CT	N	A	N	N	N	3.7	7.4
PET/MR	N	A	N	N	N	2.3	5.8

*A* affected, *Y* yes, *N* no or not affected, *I* indeterminate

### Pooled results

The consensus diagnoses based on PET/MR and PET/CT images were identical for all five patients. One discrepancy between PET/MRI and PET/CT was found in assessment of the features: for patient 2, PET/CT could not reveal a fistula that was recognized on PET/MR. MRI and PET signal was mostly congruent, except for adjacent segment bone marrow. MRI was not able to provide differentiation between reactive oedema and infected oedema signals. This led to an overestimation of infection extent by MRI as compared to PET.

Absolute SUV_max_ values varied from 2.3 to 14.0 in areas that were diagnosed as being infected. The ratio of SUV_max_ measured with PET/MRI compared to that measured with PET/CT was close to 1 (range 0.6–1.3). TBR values ranged from 4.8 to 46.7. TBR was four out of five cases highest for PET/MR, being 0.8 to 2.0 times higher than PET/CT TBR (Table 6).

**Table 6:** Pooled results

Number	Age/sex	PET/MRI and PET/CT diagnosis	Definitive diagnosis based on	PET/MRI vs PET/CT discrepancies	SUV_maxPET/MRI_ to SUV_maxPET/CT_ ratio	TBR_PET/MRI_ to TBR_PET/CT_ ratio
1	53/M	OM, 3B	N/A	None	0.7	1.0
2	49/F	OM, 3B	Clinical follow-up and surgical experience	Fistula missed on PET/CT	1.3	2.0
3	63/M	OM, 3B	Microbiology	None	1.1	1.3
4	26/M	OM, 3B	Microbiology	None	1.3	1.8
5	48/F	Osteitis	Microbiology	None	0.6	0.8

*OM* osteomyelitis, followed by the Cierny-Mader type and class

## Discussion

The value of PET/MRI in the diagnosis of osteomyelitis has been discussed in literature. The ACR and the SNMMI recognized both the musculoskeletal system and infectious disorders as potential applications for PET/MRI (Subramaniam et al. 2017). Glaudemans et al. also hypothesized that hybrid PET/MRI could improve differentiation of osteomyelitis, Charcot’s joint, and soft-tissue infections (Glaudemans et al. 2012). A simulated imaging strategy by Demirev et al. showed that combined PET and MRI imaging looks promising in diagnosing osteomyelitis (Demirev et al. 2014). Kroonenburgh et al. suggested the use of PET/MRI for diagnosis of skull base osteomyelitis, with CT added to show high-resolution bone erosion (van Kroonenburgh et al. 2018).

This case series presents the first published experience with hybrid PET/MRI in diagnosis of osteomyelitis. In all five included subjects, PET/MRI and PET/CT resulted in the same diagnosis. PET/MRI showed additional information that would not have been noticed on PET/CT in one patient. Similar to the findings by Demirev et al., we found that cases where a single modality would have provided an indeterminate assessment, the second modality of the hybrid provided additional information based on which the assessment could be determined (Demirev et al. 2014). Discordant findings between the PET image and MR or CT image were observed, as could be expected from the different physical principles of the imaging modalities. Intramedullary effects of the infection are hardly distinguishable on CT images. Although it is not directly reflected in the quantitative results, soft tissue involvement could be interpreted and delineated more accurately on PET/MRI than on PET/CT.

Beside the establishment of the diagnosis, imaging is essential to give information that allows treatment planning by defining the anatomical distribution of the infected or dead bone (Govaert et al. 2017). Although the assessment is subjective, the extent of the infection would have been overestimated based on MRI, as compared to PET/MR. This finding, which is recognized in literature, means that a surgeon would extend the region of debridement unnecessarily when solely relying on MRI (Lee et al. 2016). Oedema gives a high signal on T2-weighted MR images that is a combination of the active infectious process and reactive oedema around it (Lee et al. 2016). FDG accumulation on the other hand is directly correlated to metabolic activity of the tissue, which is highest in the active infection. In surgical intervention for osteomyelitis, the amount of debridement should first be aggressive enough to ensure total removal of the infected tissue and as little as possible to limit the compromise to structural integrity. PET/MRI was able to correlate the infection site location based on FDG PET metabolic imaging clearly with soft tissue features on MRI (as is evidently depicted in Fig. 1). This information can guide the surgeon in debridement preparation. Additionally, MRI adds both soft tissue and bone reference to the PET image, whilst CT mainly adds bony landmarks. Not only does this aid in intervention in the bone, but it also helps to plan soft tissue interventions. This led to the impression that PET/MRI could be of greater incremental value in treatment planning than PET/CT.

The SUV is a quantitative measure of FDG uptake in a volume of interest, and its use is part of routine clinical practice in FDG PET oncology. SUV is not routinely used in initial infection diagnosis but can be used to objectively monitor therapy response or to correlate images between the two hybrid modalities. The PET/CT and PET/MRI scanners in our hospital are cross-calibrated every 3 months according to the EANM EARL guidelines (Boellaard et al. 2014). The measured PET/CT and PET/MRI SUV_max_ values showed no substantial differences, with a maximum difference in one patient of a factor 0.6. It has been reported before that SUV values correlate well between PET/MRI and PET/CT (Lyons et al. 2015; Kershah et al. 2013). MRI-based attenuation correction maps such as were used in the present study do not take the increased attenuation by the bone into account, but research showed that SUV_max_ values differed by only 8% in pelvic and spinal lesions when MRI-based (no bone) attenuation correction was used compared to an attenuation map with the bone (Schramm et al. 2015). Other differences in SUV values are likely influenced by the delay between PET/CT and PET/MRI scans that result in physical decay and biologic clearance or continued accumulation (van den Hoff et al. 2014; Zhuang et al. 2001; Hamberg et al. 1994).

PET TBR values are an important indication for the detectability for a lesion. TBR was in most cases significantly higher for PET/MRI than PET/CT. This was presumably related to the higher sensitivity of the PET/MRI scanner and the acquisition time that was in general longer for PET/MRI as it was extended to the duration of MRI acquisition. Another important factor is the longer incubation time, as the only patient who underwent PET/MRI before PET/CT was the only case in which PET/CT TBR was higher than PET/MRI TBR.

The suitability of an imaging modality is not only dependent on the diagnostic power of the modality. Cost and availability also have a great influence in the choice for appropriate imaging. A health technology assessment should be carried out to weigh the (additional) costs of PET/MRI against the financial benefits of possibly faster appropriate treatment, reduced morbidity, and reduced downstream testing. Moreover, radiation exposure has to be taken into account. PET/CT inherently exposes the patient to a larger dose of ionizing radiation than PET/MRI. Osteomyelitis patients in general do not have a decreased life expectancy, which makes dose optimization all the more relevant to reduce radiation-induced cancer risk. Hybrid PET/MRI can also be used to correlate increased extraosseous FDG uptake to muscular structure, indicating normal muscle activity during acquisition. With the novel hybrid modality, a patient can be scanned in a one-stop-shop with both PET and MRI leading to higher patient comfort, especially because these patients often have limited mobility. Drawbacks of PET/MRI compared to PET/CT in patient comfort are usually longer acquisitions, a smaller bore, acoustic noise, and an increased amount of contra-indications.

A limitation of the study is the status of our hospital as referral centre for complicated orthopaedic patients, which could result in a selection bias. Multifactorial processes are more commonly observed in our patient groups than in general hospitals. The lack of a contrast-enhanced MRI scan, as explained earlier, could have been a negative influence on the assessment of MR images. Although routinely used in our institute, the incremental value of gadolinium in osteomyelitis diagnosis by MRI is not without controversy in literature (Kan et al. 2010; Averill et al. 2009). In the presented series, contrast could have provided a definitive distinction in assessment of the fluid collections for patient 4. The flexible MRI receiver coils that were used are not included in the attenuation correction because their variable form and position cannot be detected by the system. Despite their design with minimal attenuation material, it is known that neglecting the receiver coil attenuation could induce local PET quantitative errors of up to 20% (Paulus et al. 2012; Eldib et al. 2016).The diagnosis of patient 2 could be regarded as a deviation from the gold standard, but microbiologic cultures are not infrequently negative in patients with osteomyelitis that has been confirmed by other techniques (Lankinen et al. 2017). It is expected that histopathological analysis could have had confirmed osteomyelitis, but in our hospital, this is only performed if a malignancy is suspected.

## Conclusion

In this small population of suspected chronic osteomyelitis patients, FDG PET/MRI led to the same diagnosis and provided at least the same diagnostic information as PET/CT. The results additionally suggest that PET/MRI is a more valuable modality for surgical planning. Infection site location based on metabolic imaging by FDG PET can clearly be correlated with soft tissue features on MRI, which is paramount for debridement planning. Aside from adequate diagnosis, this hybrid imaging modality has a reduced patient radiation dose. Based on the presented findings, our institute will start with a trial period to use PET/MRI as the standard modality of choice for osteomyelitis. A prospective study on a larger patient cohort should be performed to statistically compare sensitivity and specificity of this modality to others. In the future, we expect to report more results from our experience with hybrid PET/MRI in (chronic) osteomyelitis patients.

## Abbreviations

ACR: American College of Radiology
CRP: C-reactive protein concentration
CT: Computed tomography
ESR: Erythrocyte sedimentation rate
FDG: 2-[18F]-fluoro-2-deoxy-D-glucose
FOV: Field of view
METC: Medical-Ethical Review Committee
MR: Magnetic resonance
MRI: Magnetic resonance imaging
MTP: Metatarsophalangeal
NEMA: The Association of Electrical Equipment and Medical Imaging Manufacturers
OSEM: Ordered subset expectation maximization
PET: Positron emission tomography
SNMMI: Society of Nuclear Medicine and Molecular Imaging
SPECT: Single-photon emission tomography
STIR: Short T1 inversion recovery
SUV: Standardized uptake values
TBR: Target-to-background ratio
TSE: Turbo spin echo
WBC: Leukocyte count
